# Editorial: Epigenetic regulation of the musculoskeletal system in health, disease, and aging

**DOI:** 10.3389/fendo.2023.1143210

**Published:** 2023-01-24

**Authors:** Katarzyna Goljanek-Whysall, Simon Tew, Mandy J. Peffers, Ioannis Kanakis

**Affiliations:** ^1^ Department of Musculoskeletal & Ageing Science, Institute of Life Course & Medical Sciences, Faculty of Health & Life Sciences, University of Liverpool, Liverpool, United Kingdom; ^2^ Department of Physiology, School of Medicine and REMEDI, CMNHS, NUI Galway, Galway, Ireland; ^3^ Chester Medical School, University of Chester, Bache Hall, Countess View, Chester, United Kingdom

**Keywords:** epigenetics, miRNAs, non-coding RNAs, musculoskeletal aging, osteoporosis

Epigenetic regulation of the musculoskeletal (MSK) system is a rapidly growing field which has gained the interest of researchers over the last two decades. The (patho)physiology of different systems, including the MSK system, is controlled by epigenetic alterations, such as DNA and chromatin modifications but also microRNAs and, in a wider context, non-coding RNAs. Molecular mechanisms and, consequently, cellular functions and differentiation can be altered by epigenetic events leading to MSK disorders such as osteoporosis and osteoarthritis. Therefore, research on the epigenome can contribute to our understanding not only of the molecular/cellular pathways that result in a specific MSK disease, but also in unravelling the physiology of the MSK system. We established this Special Issue to collect studies that focused on the epigenetic effects on the MSK system in health, disease and ageing.

The first article by Ding et al. investigated the role of miR-214-3p in ankylosing spondylitis (AS). The authors reported that fibroblasts isolated from patients with AS and femoral neck fracture expressed low levels of miR-214-3p in an osteogenic environment *in vitro*, resulting in increased osteogenic capacity. This was reversed by miR-214-3p overexpression and could be due to the regulation of the BMP2-TGFβ pathway since BMP2 is a gene target of miR-214-3p. Bioinformatic analysis showed that overexpression of BMP2 resulted in increased levels of downstream molecules including BMPR2, p-Smad5/Smad5 and OCN, while the opposite effect was demonstrated following BMP2 silencing. Thus, this study demonstrated that miR-214-3p could provide a new perspective in AS treatment by preventing osteogenic differentiation of AS fibroblasts through inhibition of the BMP-TGFβ axis.

In the study by Ali et al., the authors used an integrative computational biology approach to describe cell-specific roles of non-coding RNAs (ncRNAs) in arthritis using published articles that reported ncRNAs effects in human osteoarthritic (OA) chondrocytes and human fibroblast-like synoviocytes (FLS) from rheumatoid arthritis (RA) patients. They described that individual miRNAs, lncRNAs, and circRNAs in OA chondrocytes can form networks that can regulate multiple molecular pathways and genes. Furthermore, it was shown that interactions between ncRNAs can result in the inflammatory phenotype and proliferation of FLS contributing to the RA pathology. Importantly, this work highlighted challenges that can be encountered when preforming such analyses and the expediency of *in silico* approaches as a step prior to laboratory experiments and translational research.

Zhao et al. reported a novel panel of six hub genes that could serve as a diagnostic/prognostic tool for osteoporosis. In this work, datasets were retrieved from the GEO database and weighted gene co-expression network analysis (WGCNA) was performed while differentially expressed genes (DEGs) between osteoporotic and healthy samples were also analysed. This gene set (*MYC, VEGFA, CSF1R, S100B, APOE* and *FGF13*) resulted from an integrated analysis of a training set and the authors tested this set in five different datasets providing a comprehensive approach. Thus, this study reveals a novel gene signature that could be use in early diagnosis of osteoporotic patients.

As a wider inclusion, the fourth article by Lai et al. provided an analysis aiming to explore the characteristics of randomized control trials (RCTs) in osteoporotic middle-aged and older patients registered on the International Clinical Trials Registry Platform (ICTRP). It was found that recent RCTs were conquered by retrospectively registered and open-label trials. The vast majority of the studies lacked available results and associated publications highlighting quality gap in this field.

We hope that that this collection of articles contributes to the current knowledge and provides new insights in future aspects for unravelling the effects of epigenetics and gene expression in the MSK system. We believe that this Research Topic poses new research questions and generates interest for relevant basic and translational studies in the future.

## Author contributions

All authors listed have made a substantial and intellectual contribution to the work and consented to be published.

